# Q-Rich Yeast Prion [*PSI*^+^] Accelerates Aggregation of Transthyretin, a Non-Q-Rich Human Protein

**DOI:** 10.3389/fnmol.2018.00075

**Published:** 2018-03-13

**Authors:** Meenakshi Verma, Amandeep Girdhar, Basant Patel, Nirmal K. Ganguly, Ritushree Kukreti, Vibha Taneja

**Affiliations:** ^1^Genomics and Molecular Medicine, Institute of Genomics and Integrative Biology, Council of Scientific & Industrial Research (CSIR), New Delhi, India; ^2^Department of Research, Sir Ganga Ram Hospital, New Delhi, India; ^3^Department of Biotechnology, IIT Hyderabad, New Delhi, India

**Keywords:** amyloid aggregation, cross-seeding, transthyretin, Sup35 protein, yeast prion [*PSI*^+^]

## Abstract

Interactions amongst different amyloid proteins have been proposed as a probable mechanism of aggregation and thus an important risk factor for the onset as well as progression of various neurodegenerative disorders including Alzheimer's, Parkinson's, Huntington's, and Amyotrophic Lateral Sclerosis. Evidences suggest that transthyretin (TTR), a plasma protein associated with transthyretin amyloidosis or familial polyneuropathy (FAP) interacts with heterologous amyloid proteins including amyloid beta and islet amyloid polypeptide. In addition, recent clinical studies have revealed the presence of systemic polyneuropathy associated with FAP mutations in patients with spinocerebral ataxia, amyotrophic lateral sclerosis, and new familial systematic prion disease. Hence, it is important to investigate the interactions amongst different amyloid proteins to gain better insight into the pathology of amyloid disorders. Yeast has been an excellent model system to study interaction/ cross-seeding between heterologous amyloid proteins, more because of presence of endogenous yeast prions. Here, we examined interactions of non-glutamine (non-Q)-rich transthyretin, with glutamine (Q)-rich yeast prion protein Sup35. We established aggregation of an engineered double (F87M/L110M) mutant M-TTR-GFP in yeast. This mutant is monomeric and readily formed aggregates compared to WT-TTR-GFP in yeast at acidic pH. Interestingly, aggregation of M-TTR-GFP was significantly enhanced in presence of [*PSI*^+^], an endogenous prion form of Sup35. Different variants of [*PSI*^+^] seeded M-TTR-GFP with different efficiencies and curing of [*PSI*^+^] (losing the prion form) in these strains reduced aggregation. Moreover, overexpression of prion domain of Sup35 fused to RFP (NM-RFP) also increased M-TTR-GFP aggregation. M-TTR-GFP and NM-RFP aggregates co-localized in perivacuolar and juxtranuclear region. Sup35 protein was even immunocaptured in M-TTR-GFP aggregates. However, M-TTR-GFP overexpression did not induce Sup35 aggregation. Thus, it appears to be a unidirectional interaction between these two amyloid proteins. However, no affect on M-TTR-GFP aggregation was observed due to another yeast prion, [*PIN*^+^]. Our findings thus show the molecular interaction of transthyretin with yeast prion and support that sequence similarity is not the prime requirement for heterologous amyloid interactions.

## Introduction

Transthyretin amyloidosis (ATTR) is one of the most common forms of hereditary systemic amyloidosis caused due to abnormal accumulation of transthyretin protein (TTR) in various organs/tissues. More than 100 point mutations in TTR have been associated with the disease. Even the wild type TTR has an intrinsic propensity to form aggregates and cause late onset of the sporadic form of ATTR. TTR is a tetramer in its native state and the point mutations destabilize the tetramer into monomers which undergo conformational changes (Hammarström et al., 2002). These misfolded monomers self-assemble to form a seed or nuclei during the lag phase. This seed serves as a template to recruit more misfolded monomers/conformers and catalyze its own polymerization. Therefore, the formation of nuclei or seed is considered as the time-limiting step in the process of fibrillization. The introduction of preformed “seed” can reduce or eliminate the lag phase and accelerate fibrillization. Evidences suggest that this “seed” could be homologous or heterologous in nature (Jarrett and Lansbury, 1993; Morales et al., 2009, 2013). The evidence for cross seeding of TTR was first suggested by an *in vitro* experiment, where addition of IAPP fibrils enhanced TTR aggregation (Westermark and Westermark, 2008). Further, several reports showed interaction of TTR with other amyloid proteins including Aβ (Schwarzman et al., 1994; Choi et al., 2007; Wati et al., 2009) and α-synuclein (Guerreiro et al., 2012). Recently, a new familial form of human prion disease defined as PrP systemic amyloidosis has been reported to show clinical overlap with familial amyloid polyneuropathy, which is known to be associated with TTR mutations (Mead et al., 2013; Mead and Reilly, 2015).

Yeast has served as an excellent model system to investigate homologous and heterologous seeding amongst amyloid proteins. Interestingly, yeast has endogenous prions (Wickner, 1994; Wickner et al., 1995; Patino et al., 1996; Liebman and Derkatch, 1999; Du et al., 2008; Patel et al., 2009), which interact with heterologous prions and other amyloid proteins, thus providing the proof of concept of cross-seeding hypothesis. Endogenous glutamine-rich (Q-rich) yeast prions promote or inhibit induction of other Q-rich yeast prions (Derkatch et al., 2001; Schwimmer and Masison, 2002; Bradley and Liebman, 2003; Kryndushkin et al., 2008; Yang et al., 2013; Du and Li, 2014; Ripaud et al., 2014). In addition, yeast prions have been shown to promote aggregation of other polyQ proteins such as huntingtin, a human amyloid protein (Meriin et al., 2002; Derkatch et al., 2004; Zhao et al., 2012; Kantcheva et al., 2014). Increasing line of evidence now shows interactions amongst Q-rich and non-Q-rich amyloid proteins also. Non-Q-rich human insulin, Ig light chain and yeast prion Mod5 promote Q-rich Sup35 aggregation though with varying intensities (Derkatch et al., 2004; Arslan et al., 2015). Even Q-rich yeast prion [*PIN*^+^] enhanced aggregation of non-Q/N rich fungal prion protein Het-s (Taneja et al., 2007). Evidences also indicate that some of the interactions between amyloid proteins are reciprocal (both amyloid proteins can promote aggregation of each other) whereas some are unidirectional (one amyloid protein promotes aggregation of the second amyloid protein but not vice-a-versa) (O'Nuallain et al., 2004; Westermark and Westermark, 2008).

Similar to strains of mammalian prion protein (Safar et al., 1998; Jones and Surewicz, 2005), yeast prions are known to exist as biological variants, which exhibit variable phenotype, aggregation and seeding/ cross-seeding abilities (Derkatch et al., 1996; Schlumpberger et al., 2001; Uptain et al., 2001). Variants of yeast prions, [*PSI*^+^] and [*PIN*^+^], have been well studied. Distinct variants of [*PSI*^+^] have been classified as strong [*PSI*^+^] or weak [*PSI*^+^] based on different functional activity and ability to form aggregates (Derkatch et al., 1996; Zhou et al., 1999). Furthermore, these prion variants have been shown to interact differently with other amyloid proteins. Variants of the [*PIN*^+^] prion induce [*PSI*^+^] with different efficiencies (Bradley et al., 2002). These differences amongst the prion variants can probably be explained by the ability of a protein to misfold into a range of conformers giving rise to different aggregated forms (Krishnan and Lindquist, 2005; Tanaka et al., 2006; Toyama et al., 2007).

Accumulating evidences suggest interactions amongst different heterologous amyloid proteins to be one of the risk factors in the pathogenesis of amyloid disorders. Hence, it is important to investigate how presence of one amyloid disease might influence the onset and progression of another. Examining the molecular interactions between heterologous amyloid proteins will help to gain better insight into disease pathology. In this study, we created transthyretin aggregation model in yeast and examined the interaction of TTR with endogenous yeast prion proteins. We observed that Q-rich yeast prion protein Sup35 directly interacts and enhances aggregation of non-Q-rich TTR. However, similar to previous report (Derkatch et al., 2004), overexpression of TTR did not induce Sup35 aggregation indicating a unidirectional interaction among them. Our findings suggest that not just sequence similarity but conformational fold may promote interactions among heterologous amyloid proteins and hence, support the cross-seeding hypothesis for the onset of aggregation and disease progression.

## Materials and methods

### Yeast strains, media, and plasmids

Yeast strains VL1, a [*psi*^−^][*PIN*^+^]; VL2, a [*psi*^−^][*pin*^−^]; VL3, a weak [*PSI*^+^][*pin*^−^]; VL4, a strong [*PSI*^+^][*pin*^−^] in 74-D694 background (*ade1-14 ura3-52 leu2-3, 112 trp1-289, his3-200*) were a kind gift from Dr. Susan Liebman. L2723, a *SUP35* deletion strain (*sup35*Δ:LEU2) episomally expressing full length (*N-MRF*) Sup35 (pRS313-N-MRF, *HIS*) in 74-D694 was also a gift from Dr. Liebman's lab (Bagriantsev and Liebman, 2006). As described by them, a control strain was generated by replacing the pRS313-N-MRF with plasmid pRS316-MRF expressing only the C-domain of Sup35 (MRF). W303, a wild type (*leu2-3,112 trp1-1 can1-100 ura3-1 ade2-1 his3-11,15*) yeast strain was also used.

Yeast cells were grown in standard media. Rich media contained either 1 or 0.25% yeast extract, 2% peptone and 2% dextrose. Synthetic minimal media contained all amino acids except those used for selection and 2% dextrose (*SD*). For overexpression constructs dextrose was replaced with raffinose (2%) and galactose (3%) (SRaf+Gal) in minimal media. To examine the effect of pH, media pH was adjusted to 4.2 with HCl and 6.5 with NaOH. Yeast transformations were done by lithium acetate protocol (Gietz and Woods, 2002).

The plasmid, pRS316-MRF, expressing only the C-domain of Sup35 and another plasmid pNM-RFP for overexpression of prion domain (NM) of Sup35 fused to RFP were again a gift from Dr. Susan Liebman. The wild type TTR-GFP fusion construct was generated by amplifying the full length human transthyretin cDNA from pDNR-LIB clone and inserting it upstream of GFP in a centromeric plasmid, pRS316CG. WT-TTR-GFP fusion fragment was then amplified and cloned at BamHI site in pRS424-GAL1, a 2 micron expression vector to generate pWT-TTR-GFP. Plasmid pM-TTR-GFP was obtained by introducing two point mutations simultaneously; methionine substitution at phenylalanine 87 position (F87M) and methionine substitution at leucine 110 position (L110M) in WT-TTR-GFP using QuickChange site-directed mutagenesis procedure from Stratagene (Cat# 200516). A vector control (pGFP) was created by digesting pWT-TTR-GFP with SacII enzyme and subcloning the GFP fragment at SacII site in pRS424-GAL1 vector. All the clones were verified by restriction digestion and DNA sequencing.

### Quantification of amyloid aggregates in yeast by fluorescent microscopy

Different yeast strains were transformed with either TTR-GFP overexpression constructs alone or co-transformed with plasmids expressing N-MRF or MRF. Cells were grown in synthetic glucose selection media and reinnoculated in inducing media. (SRaf+Gal) TTR aggregates were analyzed after 72 h using a Nikon Ti-E inverted fluorescent microscope. For each transformant, six or more microscopic visual fields were randomly selected and cells with aggregates and diffused GFP expression were manually counted. More than 600 cells were counted per transformant and three independent transformants were analyzed for each construct.

### Determining the native state of TTR-GFP in yeast

Yeast cells overexpressing WT-TTR-GFP or M-TTR-GFP were harvested after 24 h of incubation in the inducing media and lysed using 1X lysis buffer (50 mM TrisCl, 50 mM KCl, 10 mM MgCl_2_, 5% glycerol). Cell lysates were normalized for total protein and incubated at 37 and 95°C in non-denaturing sample buffer without β-mercaptoethanol and resolved on 12% SDS-PAGE. The blot was probed using anti-GFP antibody (Cat# G6795, Sigma).

### Centrifugation assay to analyze the distribution of overexpressed WT-TTR-GFP and M-TTR-GFP in yeast

Cell lysates were normalized for total protein at 100 μg/μl and 1 mg of total protein was centrifuged at 40,000 rpm for 3 h at 4°C. Supernatant fraction was aspirated and the pellet fraction was resuspended in the same volume (100 μl) of 1X lysis buffer as the supernatant. Equal volumes of supernatant and pellet fractions were loaded. Total protein loaded was one-tenth of the normalized protein for both the samples. The total, supernatant and pellet fractions were resolved on 10% SDS-PAGE, immunoblotted and probed using anti GFP antibody.

### Determining the effect of overexpression of WT-TTR-GFP and M-TTR-GFP on cell viability in yeast

Effect of over-expression of WT-TTR-GFP and M-TTR-GFP was measured by spotting serial dilutions of exponentially growing liquid cultures. Yeast cells with WT-TTR-GFP or M-TTR-GFP were grown in raffinose media till early log phase (0.3–0.4). The cultures were normalized to 0.2 OD and 5-fold serial dilutions were prepared (5^−1^, 5^−2^, 5^−3^, and 5^−4^). 5 μl of each dilution was spotted on glucose (uninduced media) and galactose (inducing media) selection plates and incubated at 30°C for 2–3 days.

### Determining the affect of pH of the media on intracellular pH of the yeast cells

Yeast cells were grown in SD media maintained at different pH at 30°C for 24 and 36 h. Cells were harvested, washed and incubated with 500 nM of Lysotracker Red DND-99 (Cat# L7528) at 30°C for 30 min in their respective media. Cells were washed and visualized using confocal laser scanning microscope Leica TCS SP8. Images were processed using LAS X 3.1.1 software.

### Examining the expression levels of WT-TTR-GFP and M-TTR-GFP

Protein levels of the WT-TTR-GFP and M-TTR-GFP in yeast were determined by lysing the cells, normalizing the total protein and immunoblotting using anti-GFP antibody under the defined experimental conditions (different pH, yeast strain variants). GAPDH was used as the endogenous loading control.

### Examining cellular localization and interaction of TTR aggregates withc Sup35 aggregates

Yeast cells expressing M-TTR-GFP aggregates were stained with, 4,6-diamidino-2-phenylindole (DAPI), a nuclear DNA-binding dye and FM4-64, an endocytic vesicle and vacuolar membrane dye. For nuclear staining, cells were fixed with 10% formaldehyde for 2 h, washed with phosphate-buffered saline (PBS), permeabilized by incubation in 70% ethanol for 30 min at room temperature. Cells were washed again and incubated with DAPI (1 μg/ml) for 10 min and visualized. For vacuolar staining, cells were incubated with 8 μM FM4-64 in YPD for 15 min at 30°C. Cells were washed and further incubated for 60 min in fresh YPD and then visualized under fluorescent microscope.

Colocalization of M-TTR-GFP aggregates and Sup35 aggregates were monitored by co-transforming M-TTR-GFP and prion domain (NM) of Sup35 fused with RFP (NM-RFP). Colocalization was evaluated visually, quantitatively and statistically. Cells from independent transformants were analyzed under fluorescent microscope (Nikon Ti, 100X with oil immersion) for aggregates after 72 h under FITC (GFP) and TRITC (RFP) channels and images were captured separately for both the channels. Colocalization was analyzed by merging the images from two channels using NIS-Elements AR 3.2. Cells with M-TTR-GFP, NM-RFP or both M-TTR-GFP/NM-RFP aggregates were manually counted to calculate the percentage of cells showing colocalized aggregates. The degree of colocalization between the fluorophore conjugated proteins (M-TTR-GFP and NM-RFP) was quantified by calculating Pearson's correlation coefficient using Coloc2 Fiji pluggin of the ImageJ software.

### Examine the effect of TTR aggregation on Sup35 by red/white color plate assay

To determine the effect of M-TTR on aggregation of Sup35, M-TTR-GFP construct was transformed in Δ *sup35* strain maintained by either N-MRF (expressing full length Sup35 where N is the prion domain) or MRF (expressing only functional domain of Sup35). This strain also has an *ade1* mutation which encodes for a premature stop codon. Cells were grown in SRaf+Gal selection media, normalized at 0.2 OD and 5 μl of each culture was spotted on 1/4 YPD agar plates and adenine deficient (-Ade) plates. Growth on -Ade plates was monitored after incubating at 30°C for 3 days. The color saturation on YPD medium was observed after incubation at 30°C for 2 days and then at 4°C for 3 days. If Sup35 protein which is a translation termination factor is functional, it terminates the translation of *ADE1* gene at the premature stop codon which results in accumulation of a red pigment giving red coloration to yeast cells on rich media and reduced or no growth on media lacking adenine. However, if Sup35 is in the aggregated form and its function is impaired, it leads to read-through of the premature stop codon and synthesis of full length Ade1 protein. There is no accumulation of red pigment, yeast cells appear white on rich media and grow on media lacking adenine.

### Curing of yeast prions by subculturing yeast cells on guanidine hydrochloride media

Transformants expressing M-TTR-GFP in different variants of yeast prions: [*psi*^−^], weak [*PSI*^+^], strong [*PSI*^+^], [*pin*^−^], and [*PIN*^+^] were cured of the prions by subculturing the cells 4 to 5 times on YPD plate containing 5 mM guanidine hydrochloride (GuHCl) at 30°C till the colonies turned red from pink or white on YPD plates.

### Examining the interaction of M-TTR-GFP and endogenous Sup35 aggregates in yeast by co-immunoprecipitation

The imunoprecipitation was performed according to the Catch and Release kit protocol (Cat#17-500). Briefly, the protein was isolated from weak [*PSI*^+^] cells over-expressing either M-TTR-GFP fusion protein or GFP alone by standard glass bead method using 1X lysis buffer (100 mM Tris Cl, 100 mM KCl, 20 mM MgCl_2_ and 10% glycerol) and total protein was estimated by BCA pierce kit (Cat# 23225). 1 mg of total lysate was incubated with 10 μl of anti-GFP antibodies (Cat# G1544) on column containing resin for overnight at 4°C in continuous rotation. Following incubation, the resin in the column was washed twice with 1X wash buffer to remove non-specifically bound proteins. Immunoprecipitated protein complexes were eluted with hot (95°C) 1X denaturing elution buffer, resolved by 10% SDS-polyacrylamide gels, and analyzed by immunoblotting using antibodies against the Sup35 (BE4, a polyclonal antibody, was a kind gift from Susan Liebman) and GFP (monoclonal antibody, Sigma Cat# 6795).

### Statistical analysis

Statistical analysis was performed using SPSS software for Windows, version 17.0 (SPSS, Chicago, Illinois). Data was checked for normality and two-tailed *t*-test was applied to determine the statistical significance and *p*-value less than 0.05 was considered to be significant.

## Results

### An engineered double mutant TTR (M-TTR-GFP) is monomeric in yeast and forms enhanced aggregation compared to wild type TTR (WT-TTR-GFP)

**Figure 1:**
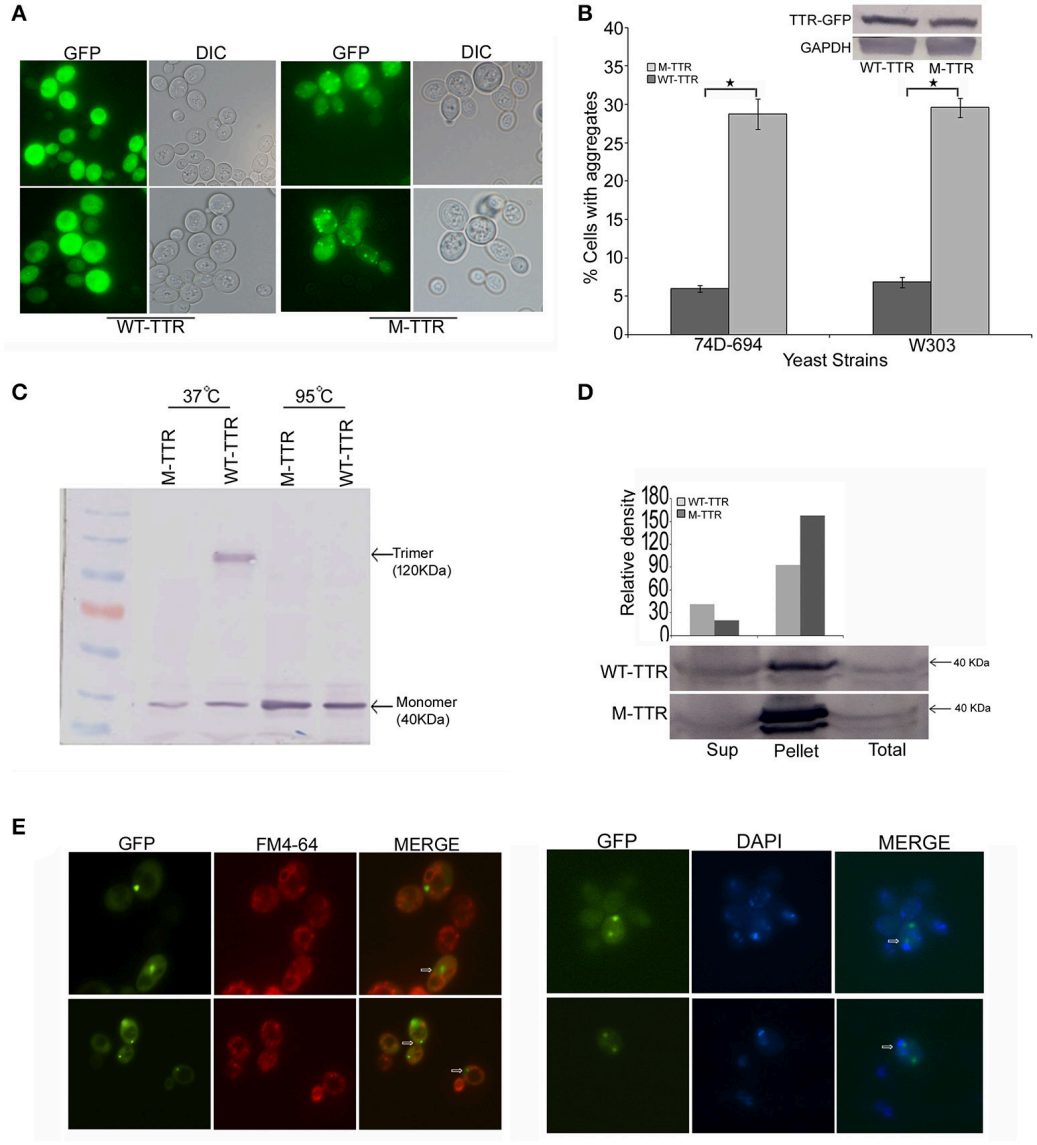
Aggregation and native state of WT-TTR and M-TTR in yeast. **(A)** The microscopic images of TTR aggregation state in yeast cells overexpressing WT-TTR-GFP and M-TTR-GFP after incubation for 72 h at 30°C. **(B)** The aggregate formation due to overexpression of WT-TTR-GFP and M-TTR-GFP was quantified in 74D-694 and W303 strains by calculating percentage of cells with aggregates. Three independent transformants and more than 600 cells were analyzed for each construct. Error bars represent standard errors of the mean of the three transformants. The significant difference in aggregation between WT-TTR-GFP and M-TTR-GFP was analyzed by using two-tailed *t*-test (^*^depicts *p*-value < 0.05). The inset on top shows the equal protein levels of WT-TTR-GFP and M-TTR-GFP as determined by immunoblotting with anti-GFP antibody. **(C)** Western blot showing the native state of WT-TTR-GFP and M-TTR-GFP in yeast. Cells expressing the two GFP fusion constructs were harvested after 24 h, lysed and the lysates were incubated under non-denaturing (37°C and without β-ME) and denaturing (95°C with β-ME) conditions. Samples were resolved on 10% SDS-PAGE and immunoblotted using anti-GFP antibody. **(D)** Centrifugation assay showing the distribution of WT-TTR-GFP and M-TTR-GFP in supernatant (Sup) and pellet fraction. Cells expressing the WT-TTR-GFP and M-TTR-GFP were harvested after 72 h and protein was isolated. Total protein was normalized before subjecting to centrifugation and equal volumes of all the fractions were loaded. The fractions were resolved on 10% SDS and probed using anti-GFP antibody. One tenth of total protein has been used as a loading control. **(E)** Cellular localization of M-TTR-GFP aggregates was analyzed by staining cells overexpressing M-TTR-GFP aggregates with FM4-64 (vacuolar) and DAPI (nuclear) dye. The images of same cells were captured under FITC and DAPI filters. The images were merged to analyze the localization of M-TTR-GFP aggregates. The white arrows in the merged panel shows the aggregates localized near the vacuole (FM4-64) and nucleus (DAPI).

To investigate TTR aggregation, wild type TTR was fused to GFP (WT-TTR-GFP) and overexpressed in yeast as described in Materials and Methods section. On overexpression of WT-TTR-GFP, most of the cells showed diffused fluorescence and only 5–7% of cells showed dot-like visible aggregates (Figures 1A,B). Since destabilization of TTR tetramer to monomer is important for aggregation, the native state of WT-TTR-GFP was analyzed. WT-TTR-GFP appeared to migrate as a higher molecular weight species at ~120 KDa (Figure 1C, lane 3). It is possible that in yeast WT-TTR-GFP exists as either trimer or tetramer that dissociates into trimer under our experimental conditions (2% SDS without β-mercaptoethanol at 37°C). These higher molecular weight species dissociated into monomer when incubated at 95°C (Figure 1C, lane 5). Another possibility is that these higher molecular weight species occur due to interaction of TTR with other cellular components. However, we reasoned it to be a trimer because trimer species have been shown to populate in the serum (Cubedo et al., 2012). Moreover, WT-TTR tetramers are formed by sequential addition of monomers; and trimer intermediates have been observed *in vitro* (Wiseman et al., 2005).

We then introduced two previously (Jiang et al., 2001) defined point mutations, F87M at the monomer-monomer and L110M at the dimer-dimer interface of WT-TTR, which destabilizes the TTR tetramer. As expected, this engineered double mutant, M-TTR-GFP, existed as monomer in yeast (Figure 1C, lane 2) and did not migrate at higher molecular weight. On overexpression of M-TTR-GFP, no aggregation of M-TTR-GFP was observed at 24 h and very minute dot-like aggregates start appearing between 36 and 48 h (Figure S1). The aggregates became clearly visible at ~72 h of incubation in inducing media under fluorescent microscope. A significant (*p* < 0.05) and approximately 5-fold increase in percentage of cells with dot-like visible aggregates compared to WT-TTR-GFP in two different (74D-694 and W303) yeast backgrounds (Figures 1A,B) was observed at 72 h. It is important to point out that more WT-TTR-GFP protein was present in the insoluble pellet fraction compared to supernatant suggesting presence of non-visible higher molecular weight species on overexpression of WT-TTR-GFP. These higher molecular weight species are the biochemical aggregates which are not visible using microscopic techniques and may include aggregation intermediates. However, M-TTR-GFP fractionated more than 2-fold in the pellet as compared to the WT-TTR-GFP (Figure 1D). It was further ensured that the increase in aggregation was not due to any difference in the expression levels of WT-TTR-GFP and M-TTR-GFP (Figure 1B, inset). The effect of aggregates on viability of yeast was monitored by dilution spotting and 7-AAD staining. No effect on growth or viability of yeast cells was observed on either episomal or stable overexpression of WT-TTR-GFP or M-TTR-GFP (data not shown). Thus, TTR-GFP aggregates appeared to be non-toxic in yeast.

Further, to visualize the cellular localization of M-TTR-GFP aggregates, cells overexpressing M-TTR-GFP were stained with FM4-64 (vacuolar staining dye) and DAPI (nucleolar staining dye). Similar to several other amyloid proteins, bigger M-TTR-GFP aggregates were observed at periphery of vacuole and smaller aggregates near the nucleus (Braun et al., 2010) (Figure 1E).

**Figure 2:**
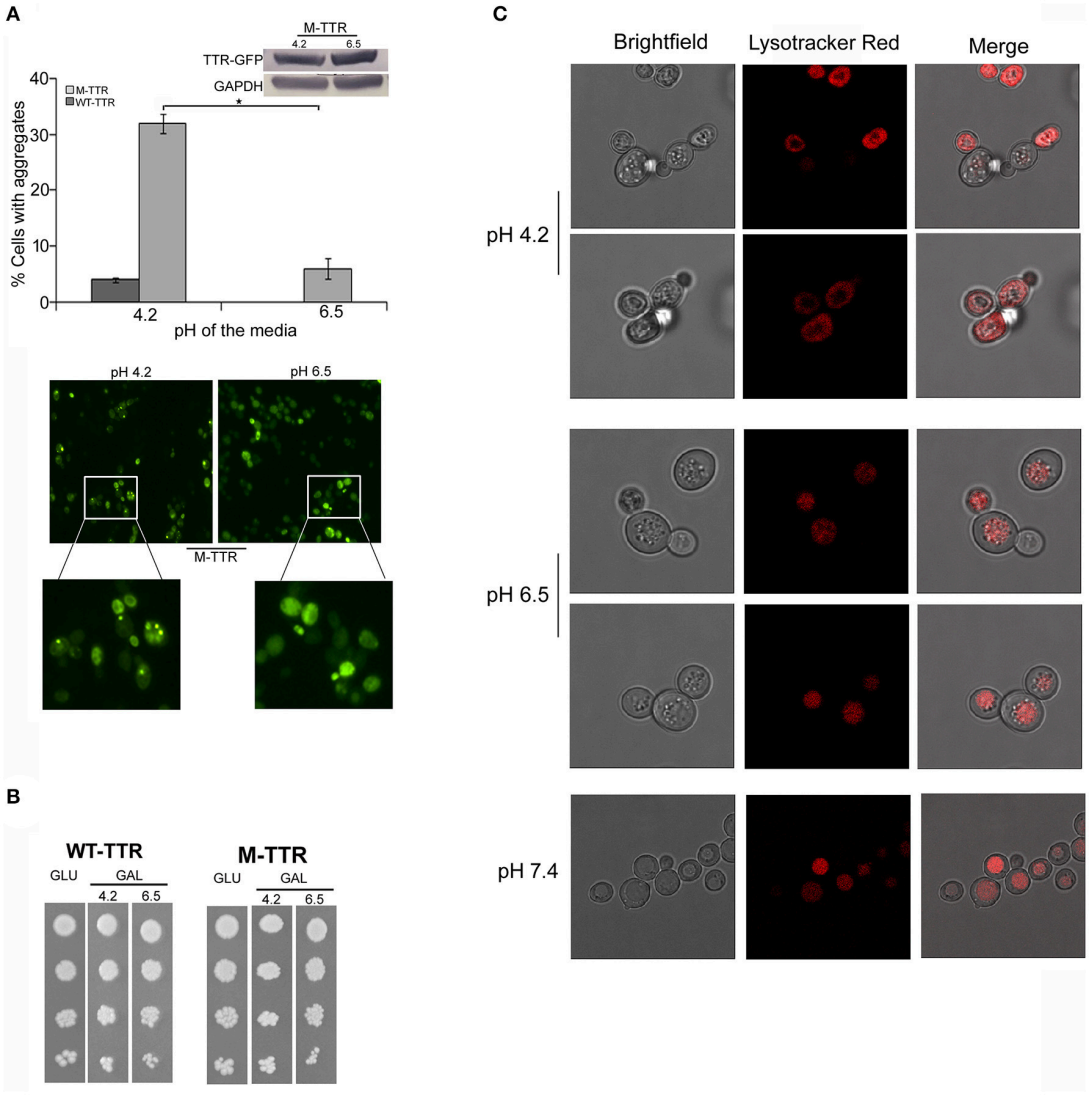
Effect of pH of the inducing media on TTR aggregation. **(A)** The effect of pH on TTR aggregation in yeast was assessed by overexpressing cells with WT-TTR-GFP or M-TTR-GFP in inducing media at pH 4.2 and pH 6.5. The percentage of cells with aggregates was counted manually under the microscope. Three independent transformants for each construct were analyzed under both conditions. Error bars in each graph represent standard errors of the mean of triplicates. To assess the significance of difference in aggregation of M-TTR-GFP at pH 4.2 and pH 6.5, two-tailed *t*-test was performed (^*^depicts *p*-value < 0.05). Equal levels of M-TTR-GFP protein at different pH were determined by immunoblotting with anti-GFP antibody. The lower panel shows a representative microscopic image of yeast cells with M-TTR-GFP aggregates at pH 4.2 and pH 6.5. **(B)** The viability of cells overexpressing M-TTR-GFP at pH 4.2 and 6.5 was determined by serial dilution spotting on inducing media (upper panel). **(C)** The microscopic images showing staining of acidic regions in the cells grown in media for 24 h with different pH using Lysotracker Red DND. Images were captured using confocal microscope Leica TCS SP8 and processed using LAS X 3.1.1 software.

The pH has been previously shown to be a factor that triggers tetramer dissociation, partial unfolding and aggregation of TTR *in vitro* (Jiang et al., 2001; Lim et al., 2013; Xue et al., 2014). It has been earlier contested and demonstrated that intracellular pH of yeast is altered by the external pH (Slavík, 1983; Thevelein et al., 1987). We also analyzed the affect of pH of media on the intracellular pH of yeast cells using Lysotracker Red probe which is an acidotropic probe and commonly used to stain acidic organelles. At external pH 7.4, vacuoles, the acidic compartment of yeast, were stained in all the cells and no cytoplasmic staining was observed. At pH 6.5, vacuoles were stained in more than 80% of the cells and cytoplasmic staining was observed in approximately 20% of the cells. Interestingly, at pH 4.2, more than 90% of the cells showed cytoplasmic staining suggesting the acidic nature of the cell interior (Figure 2C). There was no difference in Lysotracker staining after culturing for 24 or 36 h in media with different pH.

To monitor whether pH of the media has any effect on TTR aggregation in yeast, cells expressing either WT-TTR-GFP or M-TTR-GFP constructs were grown in inducing media with different pH. The percentage of cells exhibiting M-TTR-GFP aggregates was significantly reduced by 4-fold at higher pH compared to acidic pH (Figure 2A). Even, the WT-TTR-GFP aggregates were completely abolished at pH 6.5. It was also ascertained that the difference in aggregation at two different pHs was not due to different expression levels (Figure 2A, inset) or cell death as monitored by dilution spotting (Figure 2B) and 7-AAD staining (data not shown). Thus, WT-TTR-GFP or M-TTR-GFP aggregation in yeast is susceptible to acidic pH and recapitulates the *in vitro* scenario.

### [*PSI*^+^], an endogenous prion form of Sup35 enhances visible M-TTR-GFP aggregates

**Figure 3:**
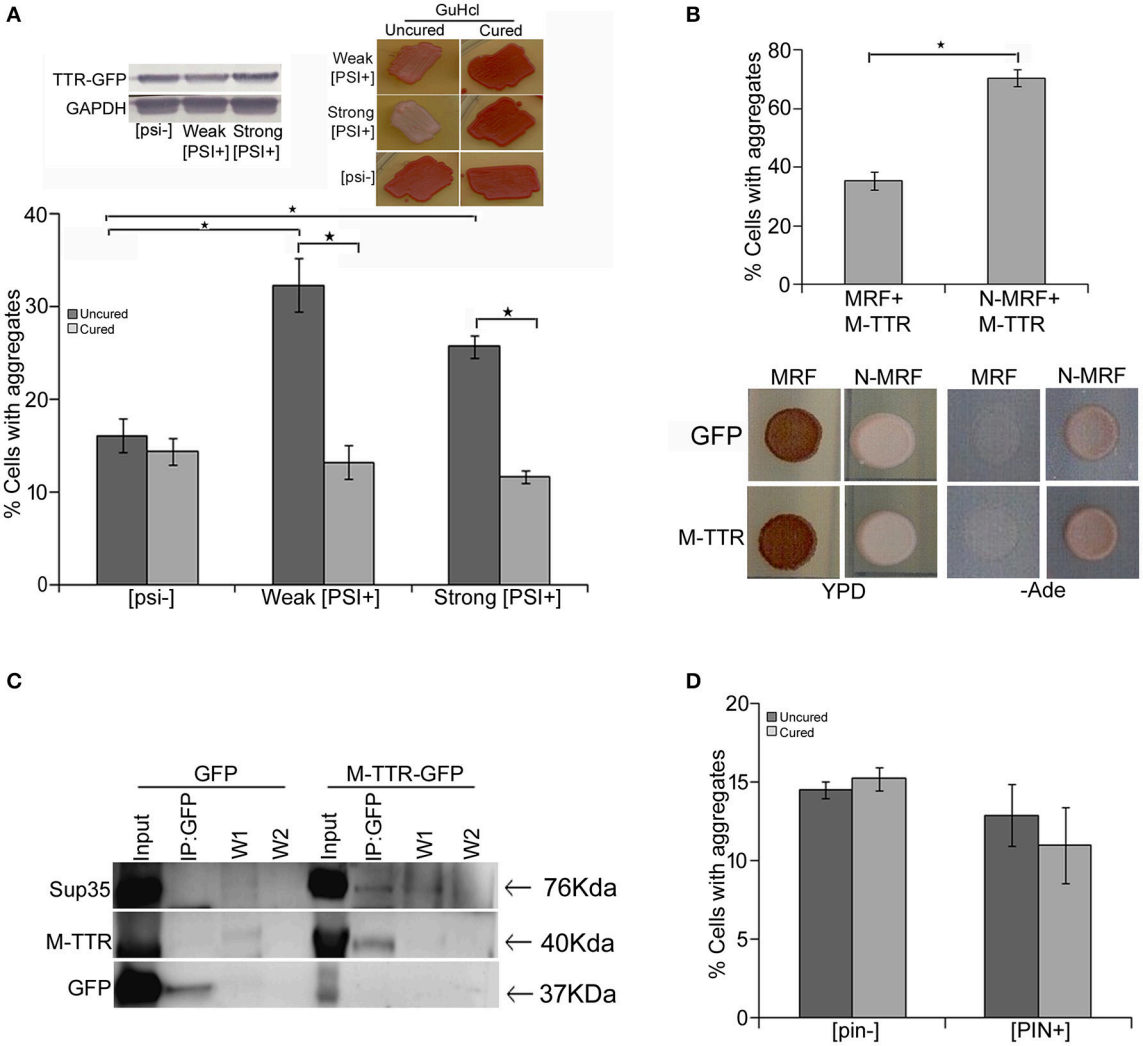
Effect of endogenous prion strains on M-TTR aggregation. **(A)** The effect of endogenous prion, [*PSI*^+^], on M-TTR aggregation was analyzed by manually counting percentage of cells with M-TTR-GFP aggregates (under the microscope) in [*psi*^−^] (non-prion) and weak and strong variants of [*PSI*^+^], depicted as uncured strains in the graph. Left inset: Equal levels of M-TTR-GFP protein in different strains were determined by immunobloting using anti-GFP antibody. M-TTR-GFP aggregation was again determined after curing the [*PSI*^+^] prion in these strains by passaging on GuHCL (right inset), depicted as cured in the graph. Error bars represent standard errors of the mean of three independent transformants. The significant difference in aggregation between [*PSI*^+^] variants and [*psi*^−^] as well as between uncured and cured strains was assessed by using two-tailed *t*-test (^*^depicts *p*-value < 0.05). **(B)** Upper panel: Percentage of cells with M-TTR-GFP aggregates were analyzed in a [*PSI*^+^] *sup35*Δ *ade1-14* strain maintained by either expression of full length (N-MRF) or the functional domain (MRF) lacking the prion (N) domain of Sup35. The strain expressing MRF could not propagate [*PSI*^+^]. Aggregation counting for all the experiments was done for atleast three independent transformants for each sample. Error bars represent standard errors of the mean of triplicates. To assess the significant difference in M-TTR-GFP aggregation between N-MRF and MRF expressing cells, two-tailed *t*-test was performed (^*^depicts *p*-value < 0.05). Lower panel: The effect of M-TTR-GFP in curing [*PSI*^+^] prion was analyzed by co-overexpressing M-TTR-GFP and N-MRF in [*PSI*^+^] *sup35*Δ *ade1-14* and spotting on rich media (YPD) and media lacking adenine (-Ade). Cells were examined for any change in coloration from white/pink (prion) to red (non-prion) on rich media and growth on -Ade media. M-TTR-GFP co-expressed with MRF (soluble functional domain of Sup35) and GFP alone co-expressed with MRF as well as N-MRF were used as controls. **(C)** Direct interaction of heterologous aggregates was examined by pull down of Sup35 with M-TTR-GFP aggregates. A weak [*PSI*^+^] strain overexpressing M-TTR-GFP fusion protein were lysed and incubated with anti-GFP antibody conjugated beads in a column. Cells overexpressing GFP were used as a control. Total protein of M-TTR-GFP and GFP lysates were normalized before incubation with the anti-GFP beads. The incubation complexes were washed twice (W1 and W2) and co-precipitated protein was eluted (IP:GFP) and resolved on 10% SDS-PAGE. Blot was probed with anti-GFP and anti-Sup35 (BE4) antibodies. Input fraction is 100 μg of the total protein lysates. **(D)** The effect of endogenous yeast prion [*PIN*^+^] on M-TTR aggregation was also analyzed by counting the percentage of cells with M-TTR-GFP aggregates in [*pin*^−^] (non-prion) and [*PIN*^+^] (prion) form of Rnq1. Aggregation counting was done for three independent transformants for each. Error bars represent standard errors of the mean of triplicates. The significance of difference in M-TTR-GFP aggregation between [*PIN*^+^] and [*pin*^−^] samples was analyzed by using two-tailed *t*-test (^*^depicts *p*-value < 0.05).

Endogenous yeast prions have been suggested to interact with other amyloid proteins. [*PSI*^+^] is a phenotypic trait of endogenous prion form of Sup35p and exists as different variants in yeast (Uptain et al., 2001) whereas [*psi*^−^] represents the non-prion form of Sup35p. In order to determine the interaction of TTR with yeast prion protein Sup35, we examined the effect of variants of [*PSI*^+^] on M-TTR-GFP aggregation. A significant (*p* < 0.05) 2-fold increase in cells with visible M-TTR-GFP aggregates was observed in weak [*PSI*^+^] and more than 1.5-folds in strong [*PSI*^+^] as compared to [*psi*^−^] strain (Figure 3A, dark gray bars). Again, the difference in aggregation was not due to any significant change in expression levels of M-TTR-GFP in the different strains (Figure 3A, left inset).

To confirm that the enhanced aggregation of TTR is solely due to presence of [*PSI*^+^], endogenous prion was cured from these transformants by passaging 8–10 times on guanidine hydrochloride (GuHCl) plates. Curing of [*PSI*^+^] leads to the elimination of prion and was monitored by change in coloration from white/pink ([*PSI*^+^]) to red as in ([*psi*^−^]) strain (Figure 3A right inset). After curing of the prion, the expression of M-TTR-GFP was re-induced in these [*psi*^−^] cells. Interestingly, M-TTR-GFP aggregation was remarkably reduced on losing the prion form of Sup35. (Figure 3A, light gray bars). This data indicates that yeast prion, [*PSI*^+^] is able to promote M-TTR-GFP aggregation.

Additionally, we overexpressed M-TTR-GFP in another [*PSI*^+^] *sup35*Δ *ade1-14* strain maintained by full length Sup35 expressed on a plasmid (N-MRF). The control non-prion form of this strain was maintained by expressing only the functional domain (MRF) but lacking the prion (N) domain of Sup35. Even in this strain background, a significant (*p* < 0.05) 2-fold increase in cells with visible aggregates of M-TTR-GFP was observed in the presence of aggregated N-MRF [*PSI*^+^] as compared to soluble MRF strain (Figure 3B, upper panel). This again supported the interaction amongst these two heterologous amyloid proteins lacking any sequence similarity. We further exploited this experimental set up to examine if overexpression of M-TTR-GFP cures [*PSI*^+^]. On overexpressing GFP alone, N-MRF [*PSI*^+^] strain is white or pink in color on YPD media and grows on -Ade media whereas cells expressing the functional, non-aggregated MRF domain of Sup35 are red on YPD and do not grow on -Ade media (Figure 3B, lower panel). On overexpression of M-TTR-GFP, however, no change in the color of the N-MRF expressing cells on rich media or growth on -Ade media (Figure 3B, lower panel) was observed. Thus, it appears that M-TTR-GFP is not able to cure the yeast prion, [*PSI*^+^].

To further verify whether these two heterologous amyloid aggregates are merely sharing the common cellular space or they exhibit any direct interaction, M-TTR-GFP fusion protein was overexpressed in a weak [*PSI*^+^] strain and M-TTR-GFP aggregates were pulled down using anti-GFP antibody. Sup35 protein co-immunoprecipated with M-TTR-GFP aggregates but was not pulled down from the cells overexpressing GFP alone as the control (Figure 3C). This observation suggests that Sup35 directly interacts with M-TTR aggregates.

In addition to [*PSI*^+^], another yeast prion [*PIN*^+^] was also examined for its effect on M-TTR aggregation. [*PIN*^+^] is the prion form of Rnq1 and has been shown to induce [*PSI*^+^] and Htt aggregation in yeast. However, we did not observe any significant difference in the percentage of cells with M-TTR-GFP aggregates in [*PIN*^+^] and [*pin*^−^] cells (Figure 3D).

### Overexpressed prion (NM) domain of Sup35 enhances M-TTR-GFP aggregation

**Figure 4:**
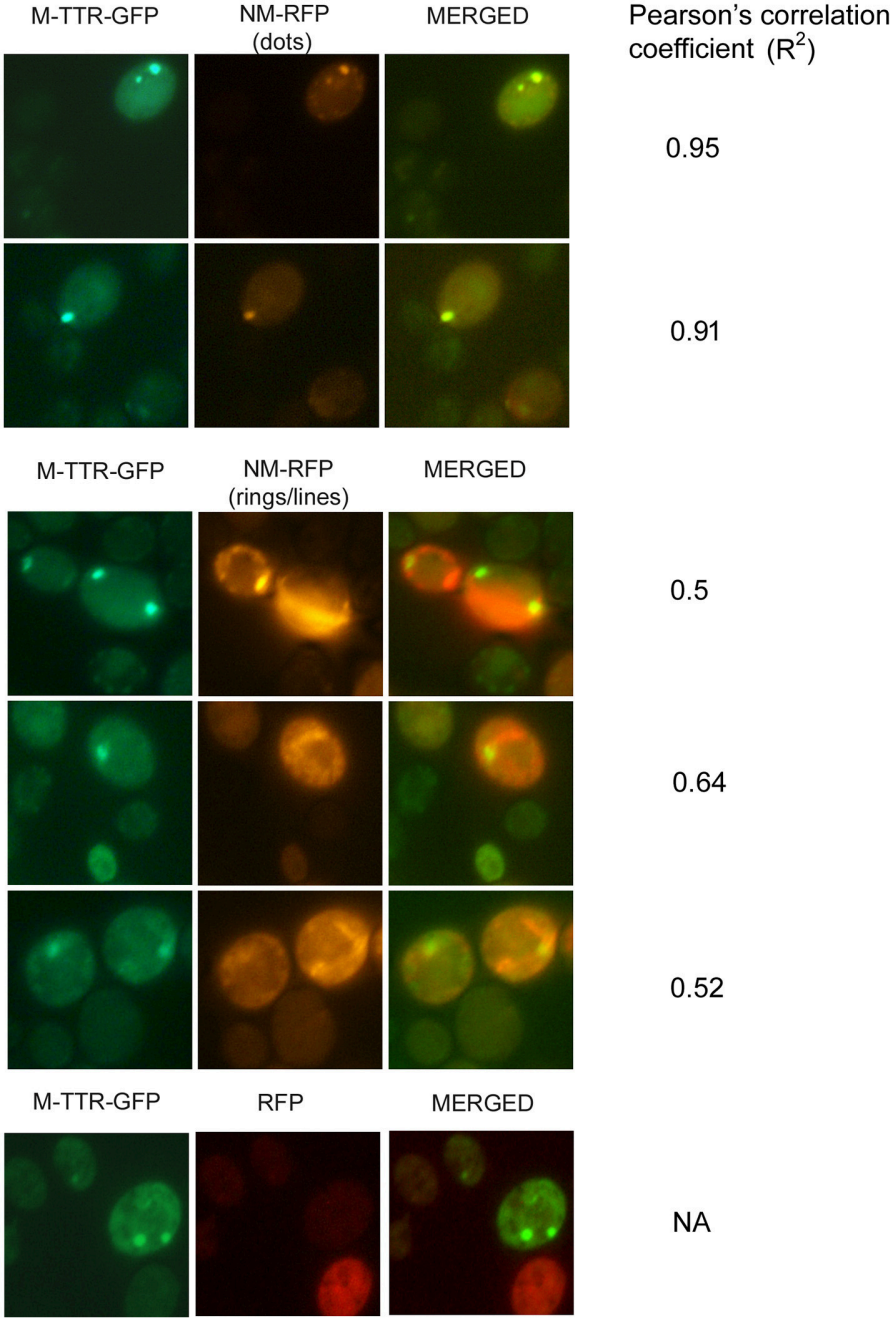
Colocalization of M-TTR-GFP and Sup35 aggregates. The microscopic images showing colocalization of M-TTR-GFP dots and Sup35 dots-, lines-, ring/mesh- like aggregates on co-overexpression of M-TTR-GFP and NM domain of Sup35 fused to RFP (NM-RFP) in yeast cells. Images were captured under FITC (GFP) and TRITC (RFP) channels and merged using NIS-Elements AR3.2 software. Pearson's correlation coefficient was determined using Coloc2.

To verify the inducing effect of Sup35 on M-TTR aggregation, we co-overexpressed M-TTR-GFP and prion (NM) domain of Sup35 fused to RFP (NM-RFP) in a [*psi*^−^] [*PIN*^+^] strain. A 2-fold increase in M-TTR-GFP aggregation was observed in the presence of overexpressed NM-RFP (~21%) as compared to RFP (~10%). On analyzing the cells for aggregates, single, two and multiple dots were observed for M-TTR-GFP and peripheral rings, lines and dots for NM-RFP as tabulated in Table 1. In about 8% of the cells, both M-TTR-GFP and NM-RFP aggregates were present and more than 90% of these cells showed colocalization. The M-TTR-GFP dots colocalized with dot-, line- (at the ends) and peripheral ring/mesh-like aggregates of NM-RFP (Figure 4). The Pearson's correlation coefficient (*R*^2^) ranged from 0.5 (for rings and lines) to 0.95 (for dots) and suggested a positive correlation of colocalization. No correlation was found between M-TTR-GFP and cytoplasmic expression of RFP alone (Figure 4). This co-localization again supports the probability of interaction between the two amyloid proteins. However, we again did not notice any significant increase in aggregation of NM-RFP (data not shown) due to the overexpression of M-TTR-GFP compared to GFP. This finding is consistent with the earlier report where addition of wild type TTR fibrils did not accelerate aggregation of Sup35-NM-YFP (Derkatch et al., 2004). Thus, the interaction of M-TTR and Sup35-NM appears to be unidirectional where overexpression of NM-RFP enhances M-TTR-GFP aggregation but not vice-a versa.

**Table 1:** Enumeration of colocalization of M-TTR-GFP and NM-RFP aggregates.

**% Cells with visible aggregates**		**% Cells with both M-TTR-GFP and NM-RFP aggregates**	**% Cells with co-localization**
**M-TTR-GFP aggregates** 10.2% single dot 4.5% two dots 6.5% multiple dots	21.2	8.2%	94% of the cells with both the aggregates
**NM-RFP aggregates** 3% peripheral rings 0.7% lines 7.2% dots	10.9		
			

*Total number of cells analyzed for co-localization (n) = 610*.

## Discussion

Tetramer dissociation of native TTR to monomers has been demonstrated to be the rate-limiting step for aggregation. In our study, wild type TTR fused to GFP exists majorly as trimer or tetramer in yeast and did not readily form visible aggregates, which is consistent with a previous observation by Gomes et al. (2012). Further, *in vitro* experiments have established that partial denaturation of transthyretin under acidic conditions cause structural changes, yielding a conformational intermediate that can self-assemble to form aggregates (McCutchen et al., 1993; Lai et al., 1996; Jiang et al., 2001; Lim et al., 2013). Here, we observed that an engineered monomeric variant of transthyretin (M-TTR) readily forms aggregates in yeast when cultured in acidic media. Interestingly, these aggregates diminished significantly at near physiological pH of the media. It is noteworthy that external pH has been shown to have effect on the cytoplasmic pH in yeast (Slavík, 1982, 1983; Valli et al., 2005). Our data also supports that when pH of the media is acidic, the cell interior also becomes acidic. Thus, aggregation of M-TTR in yeast recapitulated the *in vitro* aggregation (Jiang et al., 2001). We further exploited M-TTR aggregation model in yeast to examine the interaction of TTR with yeast prions.

Co-existence of different amyloid proteins and presence of overlapping clinico-pathological features can be probably explained by interaction among heterologous amyloid proteins. It is evident from past many studies that aggregates of one protein can accelerate or inhibit the *de novo* appearance of other amyloid proteins. Most of the earlier studies suggested sequence similarity to be the determining factor for interaction amongst heterologous amyloid proteins (Krebs et al., 2004). However, over the years, several evidences indicate that efficacy of cross-seeding does not correlate linearly with the sequence similarities. Mutant Aβ amyloid protein that differs at one amino acid is not able to seed aggregation of wild type Aβ40 (O'Nuallain et al., 2004). Interactions among Q-rich and non-Q-rich yeast or fungal prion proteins [(Sup35 and Mod5) and (Rnq1 and Het-s)] have also been demonstrated (Taneja et al., 2007; Arslan et al., 2015). Clinically, oligomerization of α-synuclein that is associated with Parkinson's disease has been observed in amyloid plaques in Alzheimer's (Hamilton, 2000), Huntington's (Charles et al., 2000), and ATTR (Guerreiro et al., 2012). In this study, we investigated the interaction between human TTR, a non-Q-rich amyloid protein and yeast prion protein Sup35 in the yeast model. Despite no sequence similarity, [*PSI*^+^], the endogenous prion form of Sup35 significantly enhanced aggregation of the engineered monomeric TTR-GFP. Further, variants of [*PSI*^+^] have been suggested to have different structural organizations and seed amyloid proteins with varying degrees. We also observed that different variants of [*PSI*^+^] seed M-TTR with different efficiencies. Loss of [*PSI*^+^] variants reduced the aggregation of M-TTR-GFP and thereby showed that enhanced aggregation was infact due to presence of heterologous seeds of [*PSI*^+^]. Increase in aggregation of M-TTR-GFP on overexpression of the prion (NM) domain of Sup35 further suggested the interaction between these two amyloid proteins. Our data also showed direct interaction between them as Sup35 was immunocaptured with M-TTR-GFP. Even the aggregation of WT-TTR-GFP, which showed very low aggregation, was enhanced in the presence of [*PSI*^+^] (data not shown).

Similar to Sup35 aggregates, M-TTR-GFP aggregates were also observed in the perivacuolar or juxtranuclear region in the cell. It can be debated that existing in the same cellular niche might be increasing the probability of interaction between these two heterologous amyloid proteins. However, another Q-rich yeast prion [*PIN*^+^], which is known to seed [*PSI*^+^] (Derkatch et al., 2001) and other heterologous amyloid proteins (Meriin et al., 2002; Derkatch et al., 2004; Taneja et al., 2007), despite being localized in the same spatial compartments in the cell, did not cause any significant effect on M-TTR-GFP aggregation. This brings to the possibility that resemblance of structural fold between the heterologous seed and misfolded soluble monomer of the protein to be seeded may influence the interaction and determine the seeding efficiency. Recent evidences suggest vast structural diversity among amyloid proteins and even the same polypeptide can adopt different conformation that give rise to different variants or strains as observed for prions. Hence, further structural insight may help in addressing the interaction between heterologous amyloid proteins and its role in genesis and progression of various amyloid disorders.

Additionally, our study highlights non-reciprocity in heterologous seeding. We did not observe any effect of M-TTR-GFP on Sup35 aggregation as previously shown by Derkatch et al. (2004). They observed occasional colocalization of Sup35 (NM-YFP) with WT-TTR aggregates but WT-TTR aggregates did not promote the [*PSI*^+^] induction. They stated that colocalization in the same cellular niche does not necessarily suggest interaction amongst the two amyloid proteins. However, our data suggests that this colocalization of M-TTR-GFP and NM-RFP (Sup35) results in unidirectional interaction which promotes M-TTR-GFP aggregation but not vice-a-versa. This lack of reciprocity in seeding has been documented for other amyloid proteins including Aβ and IAPP (O'Nuallain et al., 2004) and TTR and IAPP (Westermark and Westermark, 2008). Thus, we believe that the detailed structural determination of TTR and Sup35 may reveal an important aspect of cross seeding efficiencies. Further, targeting M-TTR to a different cellular compartment might help to establish that colocalization and direct interaction with Sup35 aggregates enhances M-TTR aggregation.

Thus, our findings provide evidence for interaction of transthyretin, a non-Q-rich amyloid protein with a Q-rich heterologous yeast prion protein that can be further explored for their relevance in the clinical scenario. Such interactions between heterologous amyloid proteins may explain how occurrence of one amyloid disorder could induce the development of another amyloid disorder. While our finding that Sup35 directly interacts with M-TTR aligns with the cross-seeding model, however the alternate possibility of indirect sequestration of cellular factors could not be ruled out. Since cellular pathways controlling aggregate formation appear to be conserved from yeast to mammals, therefore evaluating titration of other cellular factors during heterologous interaction might provide new approach for amyloid disease intervention.

## Author contributions

MV and VT: Conceived and designed the experiments; MV and AG: Performed the experiments; MV, AG, RK, NG, and VT: Analyzed the data; MV and BP: Interpretation of data; MV, RK, and VT: Wrote the manuscript; RK, BP, and NG: Critically reviewed the paper.

### Conflict of interest statement

The authors declare that the research was conducted in the absence of any commercial or financial relationships that could be construed as a potential conflict of interest.

## Abbreviations

TTR: transthyretin
WT-TTR: wild type transthyretin
M-TTR: monomeric transthyretin
AB: amyloid beta
GFP: green fluorescence protein
GuHCl: guanidine hydrochloride.
