# Supplementary figures and images for: Q-Rich Yeast Prion [PSI+] Accelerates Aggregation of Transthyretin, a Non-Q-Rich Human Protein
Source: Front Mol Neurosci. 2018 Mar 13;11:75. doi: 10.3389/fnmol.2018.00075 (PMC5859028; doi:10.3389/fnmol.2018.00075)

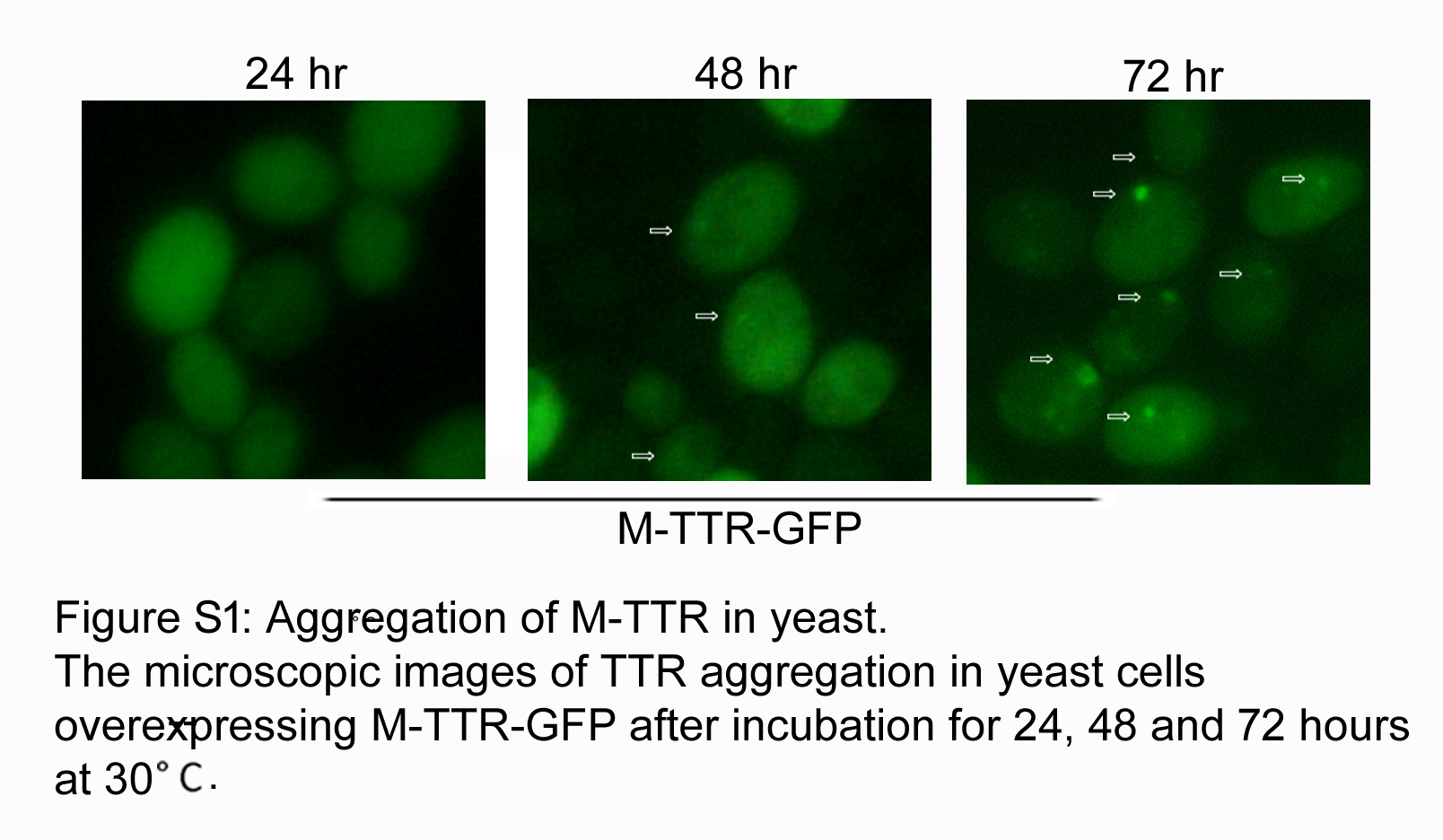

Supplement: Supplementary file 1 [file Image1.TIF]
